# Alpha Mangostin Inhibits the Proliferation and Activation of Acetaldehyde Induced Hepatic Stellate Cells through TGF-*β* and ERK 1/2 Pathways

**DOI:** 10.1155/2018/5360496

**Published:** 2018-11-14

**Authors:** Novriantika Lestari, Melva Louisa, Vivian Soetikno, Averina Geffanie Suwana, Putra Andito Ramadhan, Taufiq Akmal, Wawaimuli Arozal

**Affiliations:** ^1^Master Program in Biomedical Sciences, Faculty of Medicine, Universitas Indonesia, Indonesia; ^2^Department of Pharmacology, Faculty of Medicine and Health Sciences, University of Bengkulu, Indonesia; ^3^Department of Pharmacology and Therapeutics, Faculty of Medicine, Universitas Indonesia, Indonesia; ^4^Faculty of Medicine, Universitas Indonesia, Indonesia

## Abstract

Liver fibrosis is characterized by excessive accumulation of extracellular matrix in chronic liver injury. Alcohol-induced fibrosis may develop into cirrhosis, one of the major causes of liver disease mortality. Previous studies have shown that alpha mangostin can decrease ratio of pSmad/Smad and pAkt/Akt in TGF-*β*-induced liver fibrosis model* in vitro*. Further investigation of the mechanism of action of alpha mangostin in liver fibrosis model still needs to be done. The present study aimed to analyze the mechanism of action of alpha mangostin on acetaldehyde induced liver fibrosis model on TGF-*β* and ERK 1/2 pathways. Immortalized HSCs, LX-2 cells, were incubated with acetaldehyde, acetaldehyde with alpha mangostin (10 and 20 *μ*M), or alpha mangostin only (10 *μ*M). Sorafenib 10 *μ*M was used as positive control. LX-2 viability was counted using trypan blue exclusion method. The effect of alpha mangostin on hepatic stellate cells proliferation and activation markers and its possible mechanism of action via TGF-*β* and ERK1/2 were studied. Acetaldehyde was shown to increase proliferation and expression of profibrogenic and migration markers on HSC, while alpha mangostin treatment resulted in a reduced proliferation and migration of HSC and decreased Ki-67 and pERK 1/2 expressions. These findings were followed with decreased expressions and concentrations of TGF-*β*; decreased expression of Col1A1, TIMP1, and TIMP3; increased expression of MnSOD and GPx; and reduction in intracellular reactive oxygen species. These effects were shown to be dose dependent. Therefore, we conclude that alpha mangostin inhibits hepatic stellate cells proliferation and activation through TGF-*β* and ERK 1/2 pathways.

## 1. Introduction

Liver fibrosis is characterized by excessive accumulation of extracellular matrix including collagen tissue in chronic liver injury [1]. Liver fibrosis can be caused partly due to chronic infection of hepatitis C virus, alcohol consumption, and nonalcoholic steatohepatitis (NASH). Cirrhosis due to alcohol consumption is one of the major causes of morbidity and mortality associated with liver disease [2, 3].

Hepatic stellate cells (HSCs) play an important role as a central component in the fibrosis process induced by alcohol consumption. In normal liver, HSC is a quiescent and serves as a retinoid deposit that will differentiate into myofibroblasts when activated [4, 5]. The stimulus of HSC activation may be derived from paracrine stimulation by hepatocyte cells, Kupffer cells, and injured endothelial cells. In active HSCs, the synthesis of endogenous TGF-*β* increases significantly. The results of alcohol metabolism also increase the production of TGF-*β*. Consequently, TGF-*β* synergizes with alcohol in inducing oxidative stress, thus increasing alcohol-induced liver damage [6–8].

Activated HSCs also take part in the accumulation of extracellular matrix. Extracellular matrix within the normal liver is composed of collagen (types I, II, III, and IV) and noncollagenous glycoproteins (laminin, fibronectin, and proteoglycans). In the normal liver sinusoid, there is a basement membrane matrix which comprises laminin and collagen type IV. During the development of liver fibrosis, the extracellular matrix becomes progressively replaced by interstitial collagen (types I and III). TGF-*β* also promotes liver fibrosis by regulating Matrix Metalloproteinases (MMPs) and Tissue Inhibitor of Metalloproteinases (TIMPs) secretions. MMPs and TIMPs simultaneously regulate the balance of synthesis and degradation of extracellular matrix [9, 10]. Activated MMPs will further facilitates migratory capacity of HSCs [11].

Acetaldehyde, the product of ethanol metabolism, has a direct effect on HSC activation. Acetaldehyde increases TGF-*β* synthesis in HSC. The secreted TGF-*β* has a direct correlation with the expression of *α*-Smooth Muscle Actin (*α*-SMA) and Col1A1 [12, 13]. Acetaldehyde also acts on PKC which will further activate ERK 1/2 signal transduction pathway. TGF- *β* in HSC will also activate ERK 1/2 signaling in Smad-independent pathway. Increase in ERK levels and function will then induce cell proliferation and lead to spontaneous activation of HSC and thereafter undergoing differentiation into myofibroblasts [14, 15].

In HSC, acetaldehyde metabolism produces reactive oxygen species (ROS) [12]. ROS plays an important role in the fibrogenesis process due to alcoholism. TGF-*β* will increase ROS production and suppress antioxidant production. On the other hand, ROS will also activate latent TGF-*β* as well as inducing its expression. Hydrogen peroxide also activates the MAPK path which will then activate the signal transduction pathway ERK 1/2 [16, 17].

Alpha mangostin, a lead compound found in the fruit* Garcinia mangostana*, is thought to be able to suppress fibrogenesis process. It has been reported that alpha mangostin has antiproliferative and antioxidant activity. Previous studies have shown that alpha mangostin can decrease ratio of pSmad/Smad and pAkt/Akt in TGF-*β*-induced liver fibrosis model* in vitro* [18]. Further investigation of the mechanism of action of alpha mangostin as a antifibrosis, particularly due to alcoholic liver disease, still needs to be done. Therefore, this study aimed to analyze the mechanism of action of alpha mangostin on acetaldehyde induced liver fibrosis model on TGF-*β* and ERK 1/2 pathways.

## 2. Materials and Methods

### 2.1. Materials

Human hepatic stellate cells, LX-2, were purchased from Millipore (USA, Cat No. SCC064). Sorafenib and alfa mangostin were obtained from Santa Cruz Biotechnology (USA). Acetaldehyde, dimethylsulfoxide (DMSO), and TGF-*β*1 ELISA kit were from Sigma Aldrich (Singapore). DMEM-high glucose, FBS-heat inactivated, Penicillin/Streptomycin, and Fungizone were from Biowest (USA). High pure RNA isolation kit and Transcriptor First Strand cDNA synthesis kit were purchased from Roche (USA). Thunderbird SYBR qPCR Mix was purchased from Toyobo (Japan). Cell lysis buffer and Coomassie Plus (Bradford) assay kit were obtained from Invitrogen (USA). Primers were purchased from First Base (Singapore). Alpha smooth muscle actin antibody (PA5-19465) was purchased from Thermo Fisher (USA), GAPDH antibody was purchased from Santa Cruz Biotechnology (USA), and anti-rabbit IgG, HRP-linked antibody, ERK1/2, phospho-ERK1/2 rabbit mAb were obtained from Cell Signaling Technology (USA).

### 2.2. LX-2 Culture

Immortalized hepatic stellate cells, LX-2, were cultured using the method stated in previous study by Rahmaniah et al. [18].

### 2.3. Acetaldehyde Dose

Cells were seeded at 2 × 10^4^ per well on a 24-well plate and incubated for 24 hours in a CO_2_ incubator. Afterwards, cells were treated with acetaldehyde at various doses (0 to 200 *μ*M) and negative control (DMSO) for 24 hours. Acetaldehyde dose used for treatment with alpha mangostin was the one that produces approximately twice the cell viability over control.

### 2.4. Cell Treatment

Cells were divided into six groups and seeded at 2 × 10^6^ for each group in 10-cm culture dish as follows: two groups were the untreated cells, while the other four groups were induced by acetaldehyde 100 *μ*M for 24 h. Afterwards, the medium was changed. The first group was treated with vehicle only, while in the second group, acetaldehyde 100 *μ*M was applied. For the remaining groups, the treatments applied were as follows: acetaldehyde 100 *μ*M + sorafenib 10 *μ*M, acetaldehyde 100 *μ*M + alpha mangostin 10 *μ*M, and acetaldehyde 100 *μ*M + alpha mangostin 20 *μ*M, or alpha mangostin 10 *μ*M. After 24-hour treatment, the cells were harvested and subjected for analysis of cell viability, RNA, and protein isolation. Experiments were done at four different times in duplicate. Cell viability examination was done using trypan blue exclusion method.

### 2.5. Quantitative RT-PCR Analysis

RNA was isolated from 10^6^ cells using High Pure Isolation RNA kit (Roche) and then synthesized to cDNA in accordance with the manufacturer's protocol. The mRNA expressions of Ki-67, TGF-*β*1, and TGF-*β*1 receptor were analyzed using qRT-PCR, with *β*-actin as housekeeping gene. The primer sequences used for Ki-67, Col1A1, and *β*-actin in this study were as stated in Rahmaniah et al. [18]; primers for TIMP1, TIMP2, MMP2, and MMP9 were from Dai et al. [19]; primers for MnSOD were from Hardiany et al. [20]; primers for GPX were from Dietrich et al. [21]; and the primer sequences for TGF-*β*1 and TGF-*β*1 receptor are as follows: TGF-*β*1 Fwd: 5′-TGAACCGGCCTTTCCTGCTTCTACATG-3′; TGF-*β*1 Rev: 5′-GCGGAAGTCAATGTACAGCTGCCGC-3′; TGF-*β*1-R Fwd: 5′-TTGCTGGACCAGTGTGCTTCG-3′; TGF-*β*1-R Rev: 5′-CCATCTGTTTGGGATATTTGGCC-3′.

### 2.6. Western Blot Analysis

Western blot analysis was done using 70 *μ*g protein, with primary antibody for *α*-SMA, ERK 1/2, pERK 1/2, or GAPDH according to the method by Rahmaniah et al. [18].

### 2.7. TGF-*β*1 Concentrations

TGF-*β*1 concentrations in the cell medium were measured using Human TGF-*β*1 ELISA kit (Sigma Aldrich) in 450 nm using microplate reader, according to the manufacturer's protocol.

### 2.8. Assay of Cellular Reactive Oxygen Species

Dichlorofluorescein (DCF)-sensitive cellular reactive oxygen species (ROS) in LX-2 cells after drug treatment were measured by a fluorimetric assay with dichloro-dihydro-fluorescein diacetate (DCFH-DA) assay. In brief, 10,000 cells in 96-well plates were incubated with 6 groups of treatment as stated in drug treatment section. After treatments, medium was discarded, and the cells were washed using sterile PBS. Then, 20 *μ*M of DCFH-DA was added to the cells and incubated for 30 minutes at 37°C in the dark. Cells were then washed again using sterile PBS and ROS were measured using spectrofluorometer at 485 nm excitation and 535 nm emissions.

### 2.9. Statistical Analysis

Results were presented as mean ± SEM. Statistical analysis was performed using one-way ANOVA analysis. Statistical significance was determined at the level of p < 0.05. All the graphs were produced using GraphPad Prism 7 (USA).

## 3. Results

### 3.1. LX-2 Cell Morphology

**Figure 1:**
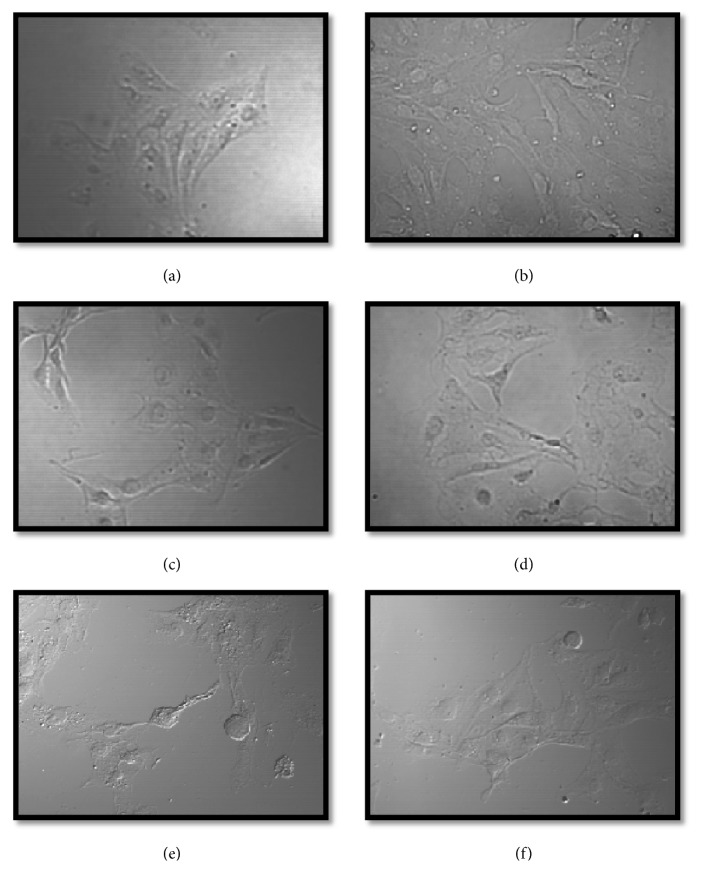
Morphological observation of LX-2 cells after treatment with (a) vehicle only, (b) acetaldehyde 100 *μ*M, (c) acetaldehyde 100 *μ*M + sorafenib 10 *μ*M, (d) acetaldehyde 100 *μ*M + alpha mangostin 10 *μ*M, (e) acetaldehyde 100 *μ*M acetaldehyde + alpha mangostin 20 *μ*M, and (f) acetaldehyde 100 *μ*M + alpha mangostin 10 *μ*M. Observation was done under confocal microscope (20 x magnification).

During the treatment, morphological observation of LX-2 cells with a confocal microscope was performed. Treatment of LX-2 cells with acetaldehyde with or without alpha mangostin did not result in any significant changes in cell morphology as shown in Figure 1.

### 3.2. Acetaldehyde Dose

**Figure 2:**
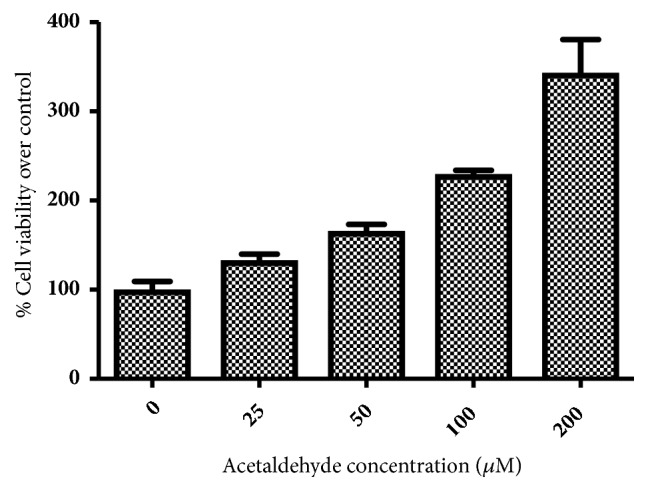
Percentage of viable cells after 24-hour treatment of LX-2 cells with acetaldehyde at 0, 25, 50, 100, and 200 *μ*M.

To establish the dose used in treatment, we determine concentration of acetaldehyde that caused approximately twice the cell viability over control. As shown in Figure 2, acetaldehyde caused increase of shown cell proliferation with increasing dose, and 100 *μ*M caused twofold cell viability over control. Therefore, we used 100 *μ*M concentration of acetaldehyde for further application.

### 3.3. Cell Proliferation

**Figure 3:**
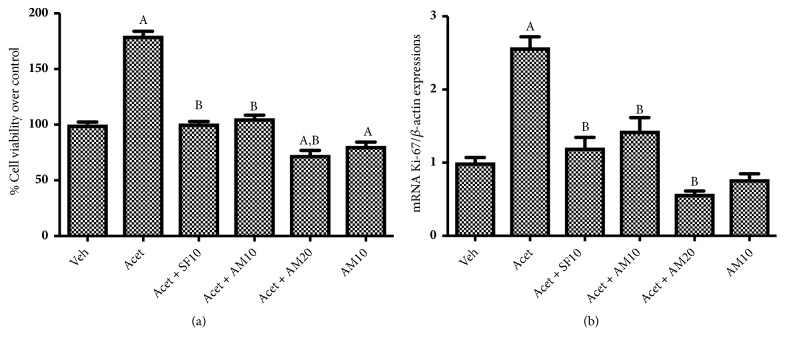
Effect of alpha mangostin in HSC proliferation as shown by (a) percentage of cell viability over control. (b) mRNA expressions of Ki67/*β*-actin expressions, after LX-2 cells were treated with alpha mangostin with or without acetaldehyde induction. Results were presented as mean ± SEM. A: p<0.05 versus untreated group, B: p<0.05 versus acetaldehyde group. Veh: vehicle only, Acet: acetaldehyde 100 *μ*M, Acet+ SF10: acetaldehyde 100 *μ*M + sorafenib 10 *μ*M, Acet + AM10: acetaldehyde 100 *μ*M + alpha mangostin 10 *μ*M, Acet + AM20: acetaldehyde 100 *μ*M + alpha mangostin 20 *μ*M, AM 10: alpha mangostin 10 *μ*M.

Acetaldehyde induction resulted in a significant increase in LX-2 proliferation as compared to vehicle only group.  Figure 3 shows that 100 *μ*M acetaldehyde significantly promotes (a) LX-2 cell viability and (b) Ki-67 mRNA expressions. Both markers were significantly suppressed by alpha mangostin in a dose-dependent manner. Treatment with alpha mangostin only resulted in a significant decrease in cell viability and proliferation.

### 3.4. Alpha Mangostin Decreased Hepatic Stellate Cells Activation as Shown by the Reduced Expression of *α*-SMA

**Figure 4:**
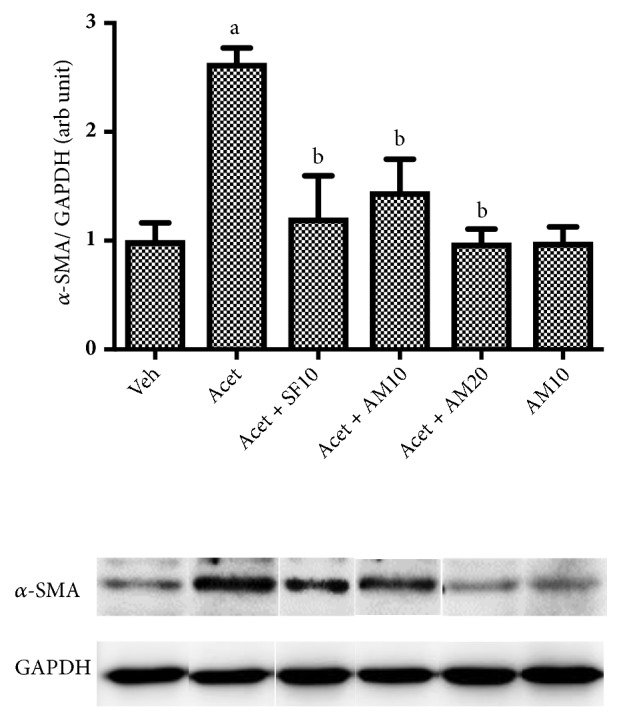
Effect of alpha mangostin in HSC activation as shown by the expression of *α*-SMA/GAPDH after LX-2 cells were treated with alpha mangostin with or without acetaldehyde induction. Results were presented as mean ± SEM. a: p<0.05 versus untreated group, b: p<0.05 versus acetaldehyde group. Veh: vehicle only, Acet: acetaldehyde 100 *μ*M, Acet+ SF10: acetaldehyde 100 *μ*M + sorafenib 10 *μ*M, Acet + AM10: acetaldehyde 100 *μ*M + alpha mangostin  10 *μ*M, Acet + AM20: acetaldehyde 100 *μ*M + alpha mangostin 20 *μ*M, AM 10: alpha mangostin 10 *μ*M.

Figure 4 shows that induction with 100 *μ*M acetaldehyde significantly increased *α*-SMA protein expression, a marker for hepatic stellate cell activation. Alpha mangostin in both dosages significantly suppressed *α*-SMA after acetaldehyde induction while alpha mangostin alone did not affect the expression of this marker.

### 3.5. Alpha Mangostin Decreased Profibrogenic Markers Col1A1, TIMP-1, TIMP-2

**Figure 5:**
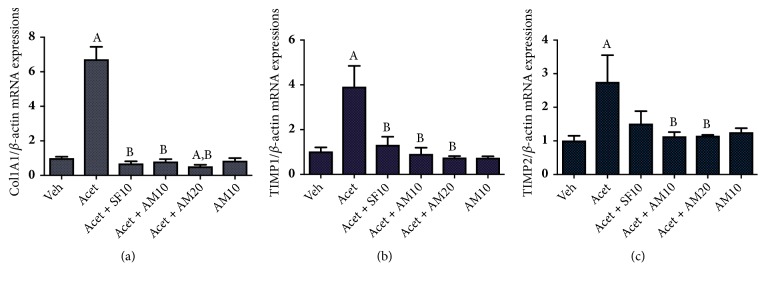
Effect of alpha mangostin on profibrogenic markers as shown by mRNA expressions of (a) Col1A1/*β*-actin, (b) TIMP1/*β*-actin, and (c) TIMP2/*β*-actin, after LX-2 cells were treated with alpha mangostin with or without acetaldehyde induction. Results were presented as mean ± SEM. A: p<0.05 versus untreated group, B: p<0.05 versus acetaldehyde group. Veh: vehicle only, Acet: acetaldehyde 100 *μ*M, Acet + SF10: acetaldehyde 100 *μ*M + sorafenib 10 *μ*M, Acet + AM10: acetaldehyde 100 *μ*M + alpha mangostin 10 *μ*M, Acet + AM20: acetaldehyde 100 *μ*M + alpha mangostin 20 *μ*M, AM 10: alpha mangostin 10 *μ*M.

Figures 5(a)–5(c) show that acetaldehyde significantly increased the expressions of several profibrogenic markers: Col1A1, TIMP1, and TIMP2. Alpha mangostin in both dosages significantly suppressed the expressions of the markers. Alpha mangostin treatment in HSCs without acetaldehyde induction did not change the expressions of the fibrogenic markers.

### 3.6. Alpha Mangostin Decreased the Expression of Hepatic Stellate Cells Migration Markers, MMP2 and MMP9

**Figure 6:**
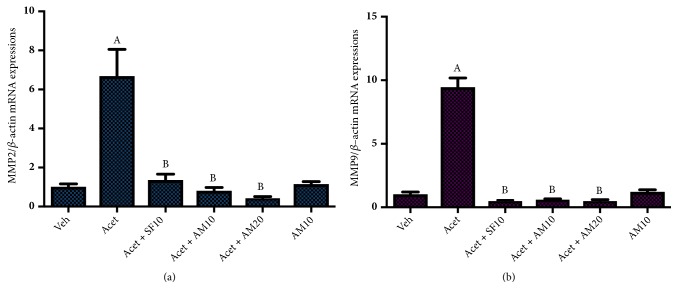
Effect of alpha mangostin on HSCs migration markers as shown by mRNA expressions of (a) MMP2/*β*-actin and (b) MMP9/*β*-actin, after LX-2 cells were treated with alpha mangostin with or without acetaldehyde induction. Results were presented as mean ± SEM. A: p<0.05 versus untreated group, B: p<0.05 versus acetaldehyde group. Veh: vehicle only, Acet: acetaldehyde 100 *μ*M, Acet + SF10: acetaldehyde 100 *μ*M + sorafenib 10 *μ*M, Acet + AM10: acetaldehyde 100 *μ*M + alpha mangostin 10 *μ*M, Acet + AM20: acetaldehyde 100 *μ*M + alpha mangostin 20 *μ*M, AM 10: alpha mangostin 10 *μ*M.

We found a significant increase in hepatic stellate cells migration markers, MMP2 and MMP9, after acetaldehyde induction. Alpha mangostin in both dosages suppressed MMP2 and MMP9 expressions after acetaldehyde induction while alpha mangostin alone did not change both markers (Figure 6).

### 3.7. Alpha Mangostin Decreased TGF-*β*1 and TGF-*β*1-Receptor mRNA Expressions and TGF-*β*1 Concentrations

**Figure 7:**
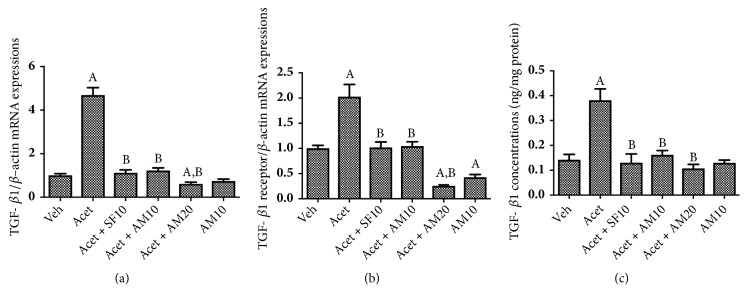
Effect of alpha mangostin in (a) mRNA expression of TGF*β*1/*β*-actin, (b) mRNA expressions of TGF*β*1 receptor/*β*-actin expressions, and (c) TGF*β*1 concentrations after LX-2 cells were treated with alpha mangostin with or without acetaldehyde induction. Results were presented as mean ± SEM. A: p<0.05 versus untreated group, B: p<0.05 versus acetaldehyde group. Veh: vehicle only, Acet: acetaldehyde 100 *μ*M, Acet+ SF10: acetaldehyde 100 *μ*M + sorafenib 10 *μ*M, Acet + AM10: acetaldehyde 100 *μ*M + alpha mangostin 10 *μ*M, Acet + AM20: acetaldehyde 100 *μ*M + alpha mangostin 20 *μ*M, AM 10: alpha mangostin 10 *μ*M.

Figures 7(a) and 7(b) show that induction with 100 *μ*M acetaldehyde significantly increased TGF-*β*1 and TGF-*β*1-R mRNA expression. The same pattern was observed with TGF-*β*1 concentrations (Figure 7(c)) as measured in culture medium. Both dosages of alpha mangostin significantly suppressed the expression of those profibrogenic markers.

### 3.8. Alpha Mangostin Decreased the Phosphorylation of ERK1/2

**Figure 8:**
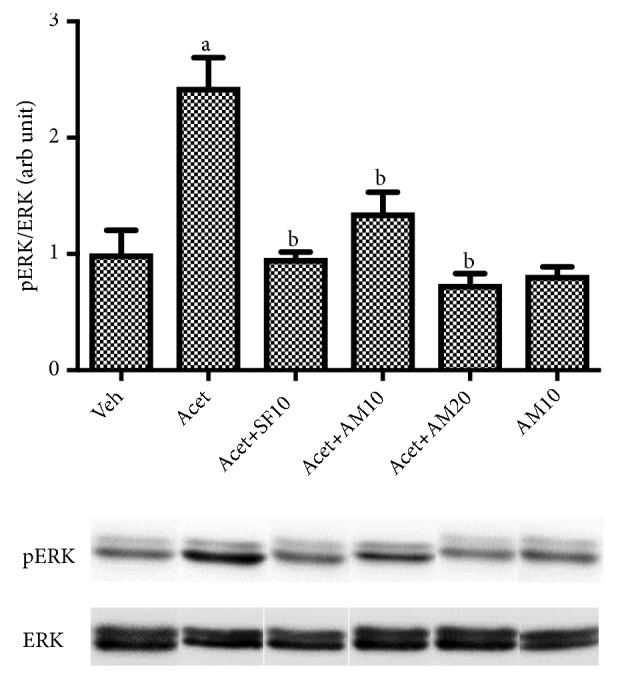
Western blot analysis for pERK/ERK1/2 expressions after LX-2 cells were treated with alpha mangostin with or without acetaldehyde induction. Results were presented as mean ± SEM. a: p<0.05 versus untreated group, b: p<0.05 versus acetaldehyde group. Veh: vehicle only, Acet: acetaldehyde 100 *μ*M, Acet + SF10: acetaldehyde 100 *μ*M + sorafenib  10 *μ*M, Acet + AM10: acetaldehyde 100 *μ*M + alpha mangostin 10 *μ*M, Acet + AM20: acetaldehyde 100 *μ*M + alpha mangostin 20 *μ*M, AM 10: alpha mangostin 10 *μ*M.

Figure 8 shows that acetaldehyde significantly increased phosphorylation of ERK1/2, while alpha mangostin at both dosages reversed ERK1/2 phosphorylation approaching untreated control (vehicle only).

### 3.9. Alpha Mangostin Decreased Intracellular ROS

**Figure 9:**
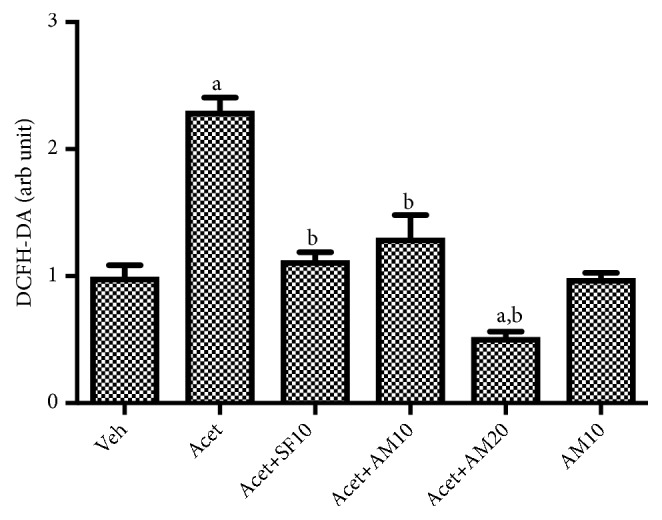
Intracellular ROS in LX-2 cells were treated with alpha mangostin with or without acetaldehyde induction. Results were presented as mean ± SEM. a: p<0.05 versus untreated group, b: p<0.05 versus acetaldehyde group. Veh: vehicle only, Acet: acetaldehyde 100 *μ*M, Acet + SF10: acetaldehyde 100 *μ*M + sorafenib 10 *μ*M, Acet + AM10: acetaldehyde 100 *μ*M + alpha mangostin 10 *μ*M, Acet + AM20: acetaldehyde 100 *μ*M + alpha mangostin 20 *μ*M, AM 10: alpha mangostin 10 *μ*M.

Figure 9 shows that induction with 100 *μ*M acetaldehyde significantly increased intracellular ROS as shown by the DCFH-DA analysis compared to vehicle only. Alpha mangostin was shown to inhibit ROS levels, while treatment with alpha mangostin only did not affect intracellular ROS.

### 3.10. Alpha Mangostin Upregulates Antioxidant Defenses mRNA Expressions, MnSOD and GPx

**Figure 10:**
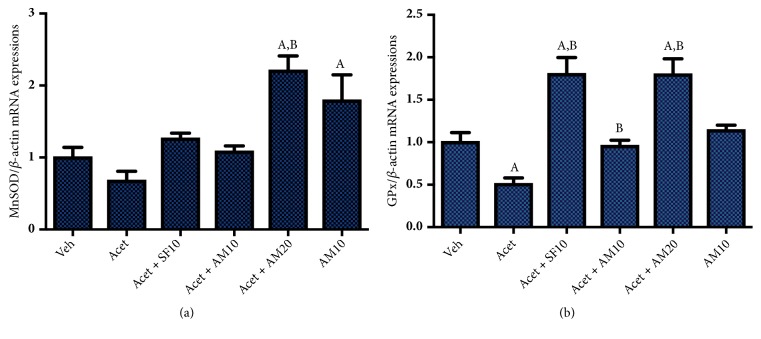
Effect of alpha mangostin on antioxidant defenses mRNA expressions of (a) MnSOD/*β*-actin and (b) GPx/*β*-actin, after LX-2 cells were treated with alpha mangostin with or without acetaldehyde induction. Results were presented as mean ± SEM. A: p<0.05 versus untreated group, B: p<0.05 versus acetaldehyde group. Veh: vehicle only, Acet: acetaldehyde 100 *μ*M, Acet + SF10: acetaldehyde 100 *μ*M + sorafenib 10 *μ*M, Acet + AM10: acetaldehyde 100 *μ*M + alpha mangostin 10 *μ*M, Acet + AM20: acetaldehyde 100 *μ*M + alpha mangostin 20 *μ*M, AM 10: alpha mangostin 10 *μ*M.

Figures 10(a) and 10(b) show that the induction with 100 *μ*M acetaldehyde decreased antioxidant defenses, as shown by the reduction of MnSOD and GPx mRNA expressions. Treatment of alpha mangostin 10 *μ*M restores the expressions of both genes, while higher dosages of alpha mangostin escalate antioxidant genes up to twofold compared to normal (vehicle group).

## 4. Discussion

Previous study has reported that alpha mangostin potently reduced hepatic stellate cells proliferation via its TGF-*β*/Smad and Akt signaling pathways, under TGF-*β* stimulation [18]. In the present study, we aimed to investigate whether alpha mangostin might have an effect on acetaldehyde induced hepatic stellate cells.

In this study there was no change in the morphology of LX-2 cells in both normal and treatment groups because it was already an active form of HSC, myofibroblast like phenotypes. The effect of acetaldehyde induced will be assessed from changes in the research biomarker.

Acetaldehyde enhances HSC proliferation as indicated by increasing cell viability and Ki-67 expression, marker of cell proliferation. Ki-67 is expressed in all cells that proliferate in both normal and cancerous cells. The marker is expressed in all active cell cycle phases (phases G1, S, G2, and M) but is not expressed in phase G0, so it can be used to describe the proliferation in a cell population [22]. It also has been known that ERK1/2 plays a role in myofibroblast proliferation. In our study we demonstrated that acetaldehyde induction affected ERK1/2 pathways by increasing phosporylation of ERK1/2. This is consistent with the research undertaken by Zhan et al. and Wang et al. finding that acetaldehyde significantly increases HSC proliferation [10, 15].

In our study, we confirmed that the administration of acetaldehyde activates HSC resulting in increased expression of *α*-SMA protein. Alpha-SMA is a component of myofibroblast cells and can be expressed in HSCs that actively form myofibroblasts-like phenotypes [23]. We also found that acetaldehyde increased all profibrogenic markers investigated, Col1A1, MMP9, MMP9, TIMP1, and TIMP2. The MMPs constitute a large family of endopeptidases, which are expressed by HSCs in response to diverse hepatic injury. MMPs play dual roles in liver fibrosis depending on the timing. Both MMP2 and MMP9 are known to degrade basement membrane collagen. Increased MMP2 and MMP9 expressions may also be profibrogenic due to their capacity to increase degradation of basement membrane collagen and replace it with interstitial collagen [10, 24]. Product of collagen degradation also affects migratory capacity of HSCs, which makes MMP2 and MMP9 good markers for HSCs migration [24].

Expressions of MMPs and TIMPs in regulating synthesis and degradation of extracellular matrix constitute a complex process. Our study had shown that TIMP1 and TIMP2 were upregulated in HSCs by acetaldehyde. This finding is consistent with the previous study by Wang et al. that showed that acetaldehyde consistently increased extracellular matrix in HSC-T6 cells and strongly promoted fibrosis development [10].

The main profibrogenic cytokines that play a role in the transformation of HSC into the active form are TGF-*β* [25]. Acetaldehyde administration increases TGF-*β* signal transduction, enhances the synthesis of endogenous TGF-*β*, and induces the expression of TCF-*β* type II receptors in HSC [26]. Acetaldehyde also increased the levels of active TGF-*β* in culture medium. The increase was correlated with an increase in *α*-SMA protein. Acetaldehyde metabolism also produces ROS. ROS plays an important role in the fibrogenesis process that occurs in alcohol-induced liver disease. TGF-*β* will increase ROS production and suppress antioxidant production. On the other hand, ROS will also activate latent TGF-*β* as well as inducing its expression [16, 17].

Previous study showed that alpha mangostin has antiproliferative activity in cancer cells. In this study, we demonstrated that alpha mangostin consistently reduced the proliferation of acetaldehyde induced HSC LX-2 as indicated by decreased cell viability, Ki-67, and pERK 1/2 expressions. Administration of alpha mangostin also reduced HSC activation as indicated by decreased expressions of *α*-SMA and Col1A1 [27]. TIMPs are secreted by activated HSCs. Our study confirmed that alpha mangostin reduces HSC activation, thus inhibiting the expression of TIMP1 and TIMP2. Alpha mangostin is also shown to inhibit HSC migration by decreasing MMP2 and MMP9 expressions. Our findings are consistent with Yuan et al. who proved that alpha mangostin inhibited migration and invasion of pancreatic cancer cells by reducing MMP2 and MMP9 expressions [28].

Alpha mangostin (dose dependent) also inhibits HSC activation by decreasing relative expression of TGF-*β* mRNA, TGF-*β*R mRNA, and active TGF-*β* level in the medium culture in acetaldehyde induced HSC. This effect is in line with* in vitro* study by Rahmaniah et al. showing that alpha mangostin can decrease TGF-*β* expression and downstream signaling transduction in TGF-*β* induced HSC mode [18].

The inhibitory effects of alpha mangostin on signal transduction ERK 1/2 are in line with studies conducted on renal cell carcinoma* in vitro* [29]. However, different results were obtained in colon carcinoma studies using HCT 116 in which alpha mangostin upregulated signal transduction of MAPK/ERK. This suggests that alpha mangostin can have different effects on different cells [30].

The administration of alpha mangostin in group without acetaldehyde induction showed a near-normal *α*-SMA protein expression. This is because there was no increase in HSC activation in the group. Alpha mangostin also decreases HSC proliferation followed by decreased profibrogenic cytokines, but has not reached significance as compared to untreated HSC.

Alpha mangostin has an antioxidant effect because it has ROS scavenging capacity [31]. The activity is confirmed by a decrease in DCFH-DA which correlates with decreased intracellular ROS levels. Alpha mangostin is also shown to upregulate antioxidant system by increasing MnSOD and GPx. ROS can activate growth factor receptors, thus activating ERK 1/2 pathway. The results showed that a stimulus that would increase the production of ROS would also activate signal transduction of ERK 1/2 in various cells. This study confirms the antioxidant activity of alpha mangostin so that decreasing ROS levels will also decrease ERK 1/2 signal transduction.

In this study using sorafenib as a positive control. Sorafenib is a receptor multikinase inhibitor currently accepted as the only effective therapy for hepatocellular carcinoma. Sorafenib has a working mechanism inhibiting ERK, VEGF, and PDGF pathways. Previous research has shown that sorafenib has potential in the treatment of liver fibrosis through inhibition of TGF *β*-induced EMT [32].

The results of this study found that alpha mangostin inhibits the proliferation and activation of acetaldehyde induced LX-2 cells through TGF-*β* and ERK 1/2 pathways. The inhibition is dose-dependent activity. This is due to an increase in effect along with the increase in dosage.

## Data Availability

The data used to support the findings of this study are available from the corresponding author upon request.
